# A Giant Renal Vein Aneurysm in a Patient with Liver Cirrhosis

**DOI:** 10.1155/2016/8751310

**Published:** 2016-09-06

**Authors:** Konstantinos Filis, George Galyfos, Ioannis Ketikoglou

**Affiliations:** ^1^Vascular Division, First Department of Propaedeutic Surgery, University of Athens Medical School, Hippokration Hospital, Athens, Greece; ^2^Department of Internal Medicine, Hippokration Hospital, Athens, Greece

## Abstract

We present an unusual case of a 40-year-old female patient with liver cirrhosis and diffuse abdominal pain. The imaging studies revealed a huge renal vein aneurysm. The patient refused any interventional management, despite the risk of possible rupture, and after a week of mild pain therapy, she was discharged. She was followed up closely, and after one year, she remains asymptomatic. Conservative management of such patients has been described before with success. However, open repair or percutaneous thrombosis of the aneurysm remains the indicated therapy, when vein patency is an issue for organ viability.

## 1. Introduction

The most common location for visceral venous aneurysms remains the portal system [1]. Venous aneurysms of the portal system are often associated with cirrhosis and portal hypertension. However, renal veins consist of an unusual location for visceral venous aneurysms (incidence < 3%), with only a small number of case reports in literature [1]. Therefore, there are insufficient data regarding optimal treatment.

The aim of this report is to present an unusual case of a patient with portal hypertension presenting with a large-sized renal vein aneurysm. Proper diagnostic and therapeutic management is discussed.

## 2. Case Report

**Figure 1:**
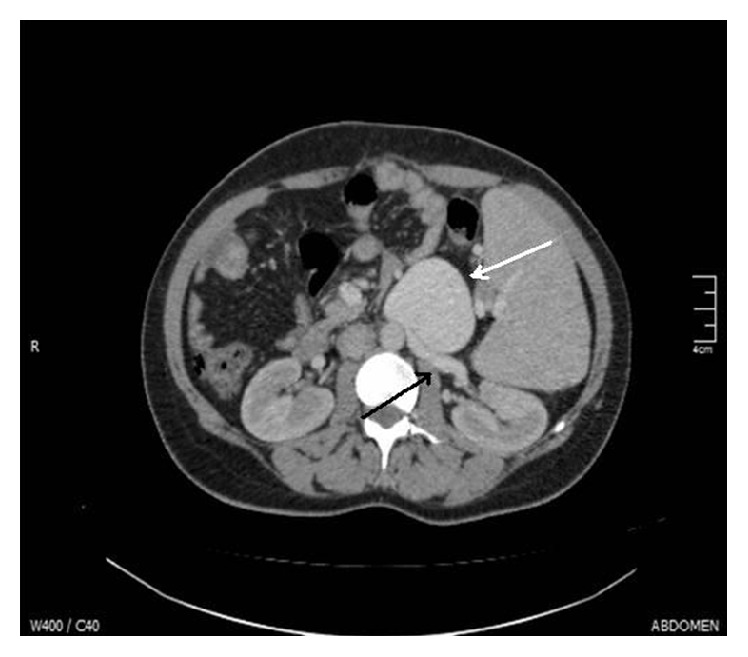
Computed angiography showing a large-sized renal vein aneurysm (white arrow) arising from the left renal vein (black arrow).

Α 40-year-old female patient with history of liver cirrhosis due to chronic hepatitis B infection presented complaining of diffuse abdominal pain. Her medical history revealed portal hypertension without ascites or encephalopathy. No acute or distended abdomen was found during physical examination. Moreover, her laboratory investigations were unremarkable. Duplex ultrasonography revealed portal vein thrombosis as well as a left renal vein aneurysm showing increased turbulent venous flow (30 cm/sec). A computed angiography followed that showed an enlarged tortuous left renal vein forming a giant saccular aneurysm (7.8 cm × 6.0 cm) (Figure 1).

A vein aneurysm resection or endovascular aneurysm embolization was recommended due to the risk of possible rupture. The patient, however, refused any intervention and decided to be followed up closely. After receiving mild pain therapy for a week, she was discharged, and after one year, she remains asymptomatic.

## 3. Discussion

Although a functional hemodynamic intrarenal disorder is observed in patients with liver cirrhosis [2], there is still no direct association confirmed between portal hypertension and aneurysm formation in renal veins. Portal hypertension remains the main cause for acquired cases of portal vein aneurysm formation [3] although cases of renal vein aneurysms are rare and discovered incidentally most of the times [4]. However, our patient had a history of portal vein thrombosis. This type of aneurysms may be asymptomatic or present with abdominal pain and other atypical symptoms when reaching a respectful size as in our case. Regarding the location, renal vein aneurysms are more common on the left side than on the right side due to hemodynamic (“nutcracker phenomenon”) and embryologic factors (formation of left renal vein due to anastomosis of the subcardinal veins) [1, 5, 6].

As far as proper management is concerned, computed tomography imaging using intravenous contrast media remains a useful tool for identifying such aneurysms and designing possible therapeutic strategies [5]. However, Doppler ultrasonography still remains a useful noninvasive imaging modality to easily identify and describe their basic features (size, flow, and location) [7]. Watchful waiting has been recommended with satisfying results although this should be preferably applied in aneurysms of smaller size [6]. When the risk of rupture is high as in our case or when aneurysms are symptomatic, repair of the aneurysms should be scheduled promptly. In these cases, first therapeutic choice remains the use of interventional percutaneous techniques aiming at aneurysm thrombosis, whereas open vascular reconstructions remain the alternative choice, when vein patency is an issue for target organ viability or the aneurysm itself causes compression to adjacent structures [8]. However, as underlined by many authors, durability of endovascular techniques needs to be confirmed in long-term follow-up of such patients [9].
